# Long-term cardiovascular impact of COVID-19 among hospitalised and non-hospitalised populations: a narrative synthesis review

**DOI:** 10.3389/fcvm.2026.1741293

**Published:** 2026-05-07

**Authors:** Nutan Maurya, Agnes Le, Gregory Melbourne, Josephine Sau Fan Chow

**Affiliations:** 1 South Western Sydney Nursing and Midwifery Research Alliance, South Western Sydney Local Health District, Sydney, NSW, Australia; 2 Ingham Institute for Applied Medical Research, Sydney, NSW, Australia; 3 Faculty of Health, University of Tasmania, Hobart, TAS, Australia; 4 Faculty of Medicine, University of New South Wales, Sydney, NSW, Australia; 5 Faculty of Medicine, Western Sydney University, Sydney, NSW, Australia

**Keywords:** cardiac dysfunction, cardiovascular complications, COVID-19, hospitalised, long COVID, non-hospitalised

## Abstract

**Introduction:**

COVID-19, initially recognised as a respiratory illness, affects multiple organ systems, including the cardiovascular system. Both hospitalised and non-hospitalised patients may experience persistent cardiac complications; however, the long-term impact across different levels of disease severity remains unclear. This review aims to summarise the existing evidence on the long-term cardiovascular impact of COVID-19, with a particular focus on differences between hospitalised and non-hospitalised patients.

**Method:**

PubMed, MEDLINE, CINAHL, and Embase databases were searched for studies published between December 2019 and January 2024 that investigated cardiovascular outcomes in long COVID. Studies were screened for eligibility, and data were extracted using a standardised form. Due to heterogeneity across the included studies, a narrative synthesis was performed.

**Results:**

Seventy-one studies were included, most of which were observational and conducted in Europe and Asia, with follow-up periods ranging from <1 to >24 months. Hospitalised patients reported more frequent cardiovascular symptoms; however, echocardiographic abnormalities were observed across all groups. Reporting of symptom severity was inconsistent. Common cardiovascular manifestations included palpitations, chest pain, fatigue, and arrhythmias. Persistent cardiac dysfunction and dysautonomia were observed regardless of hospitalisation status.

**Conclusion:**

Hospitalised patients are at higher risk of long-term cardiovascular complications, including myocardial injury, arrhythmias, and heart failure, while non-hospitalised individuals may experience subclinical cardiac changes. Vaccination appears to have a protective effect. Standardised, prospective studies are needed to clarify long-term cardiovascular risks and to guide follow-up care.

## 1 Introduction

Coronavirus disease 2019 (COVID-19) was officially declared a pandemic on 11 March 2020 (1). Initially, the disease was primarily associated with respiratory symptoms, leading to the belief that COVID-19 predominantly affected the lungs (2). However, it has since become evident that COVID-19 also significantly impacts on other organ systems, including the cardiovascular system (3). As the pandemic has progressed, research has highlighted a range of persistent complications beyond the acute phase of infection, including cardiovascular issues (4, 5).

In Australia, long COVID is defined using the National Institute for Health and Care Excellence (NICE) criteria, which include (i) “ongoing symptomatic COVID-19”—symptoms lasting more than 4 weeks—or (ii) “post-COVID-19 conditions/syndrome”—symptoms persisting beyond 12 weeks without an alternative diagnosis (6, 7). Cardiovascular disease (CVD) is a leading cause of death in Australia, with 45,392 deaths attributed to CVD in 2015 (7). The intersection of COVID-19 and CVD is of significant concern, as COVID-19 exacerbates cardiovascular risks and is associated with various short-term and long-term cardiovascular complications (8, 9). Short-term follow-up studies have identified outcomes such as ischaemic and non-ischaemic myocardial injury, cardiac dysfunction, arrhythmias, and dysautonomia (10). Long COVID has been linked to persistent cardiac rhythm disorders, including supraventricular and ventricular tachycardias, atrial fibrillation, and even complete heart block (10, 11).

Data indicate that cardiovascular symptoms are prevalent among individuals with long COVID, with up to 86% of patients experiencing symptoms such as palpitations (68%), chest pain (53%), and fainting (13%) months after infection (12). Other studies have also identified chest pain and arrhythmias as significant long-term complications (13, 14). Patients hospitalised with COVID-19 or requiring critical care have a significantly higher risk of experiencing cardiac events following infection compared with outpatients (2, 3, 15). However, cardiovascular abnormalities have also been reported in young, otherwise healthy individuals who were asymptomatic or only mildly symptomatic (9, 16, 17), as well as in those who seem to be completely recovered from the acute phase of infection (18). In a community-based study in which only 19% of participants were hospitalised during acute COVID-19, 26% showed mild heart impairment 4 months after their initial diagnosis (19). In another study of individuals with mild COVID-19, follow-up at 329 days revealed that 53% of the cohort had persistent cardiac symptoms, whereas 5% developed new symptoms; these cardiac symptoms were related to subclinical inflammatory cardiac involvement (20). Similarly, a community-based study conducted by Roca-Fernandez et al. (21), mainly involving non-hospitalised individuals, reported that cardiac abnormalities persisted in some individuals for up to 12 months after the onset of symptoms. One in five individuals showed cardiac abnormalities at 6 months, which persisted in more than half of this group at 12 months (21). Despite these findings, a comprehensive understanding of the long-term cardiovascular impact of COVID-19, particularly between hospitalised and non-hospitalised patients, remains limited. This narrative synthesis review aims to synthesise existing evidence on the long-term cardiovascular impact of COVID-19, with a specific focus on comparing outcomes between hospitalised and non-hospitalised patients. In addition, this review seeks to identify research gaps and provide recommendations for future studies to better understand and address the long-term cardiovascular consequences of COVID-19.

In this manuscript, we use the term “long COVID” consistently to describe symptoms or complications that persist following acute SARS-CoV-2 infection.

## 2 Methods

This narrative synthesis review included peer-reviewed, full-text studies published in English between December 2019 and January 2024 that investigated cardiovascular outcomes (e.g., atrial fibrillation, sinus tachycardia, ventricular arrhythmias, atrial and ventricular function, atrial flutter, cardiac arrest, pericarditis, myocarditis, acute coronary disease, myocardial infarction, ischaemic cardiomyopathy, angina, thromboembolic disorders, pulmonary embolism, deep vein thrombosis, transient ischaemic attacks, and stroke) as a part of long COVID. Studies involving participants under 18 years and non-original research (including systematic reviews, literature reviews, opinion pieces, editorials, newsletters, commentaries, correspondence, and dissertations) were excluded. A comprehensive and systematic search of PubMed, MEDLINE, CINAHL, and Embase was conducted using MeSH terms and free-text keywords related to long COVID and cardiovascular disease, applying Boolean operators; manual searching was also undertaken, and the search was re-run before final analysis. A list of search terms is provided in Supplementary Table 1.

Study screening was performed independently by at least two authors using predefined eligibility criteria, followed by full-text assessment and consensus-based resolution of any disagreements. Data were extracted using a standardised form that captured study characteristics, participant characteristics (including testing method, comorbidities, cardiovascular assessments, vaccination status, symptom severity, and hospitalisation status), and outcome measures related to long COVID cardiovascular events.

A systematic search with narrative synthesis was conducted; reported outcomes and findings were synthesised and grouped into specific themes identified by the authors. Results are presented in accordance with the described domains and subgroup analyses. We could not perform a meta-analysis due to heterogeneity in study design, follow-up duration, and variation in the outcomes of interest.

## 3 Results

**Figure 1:**
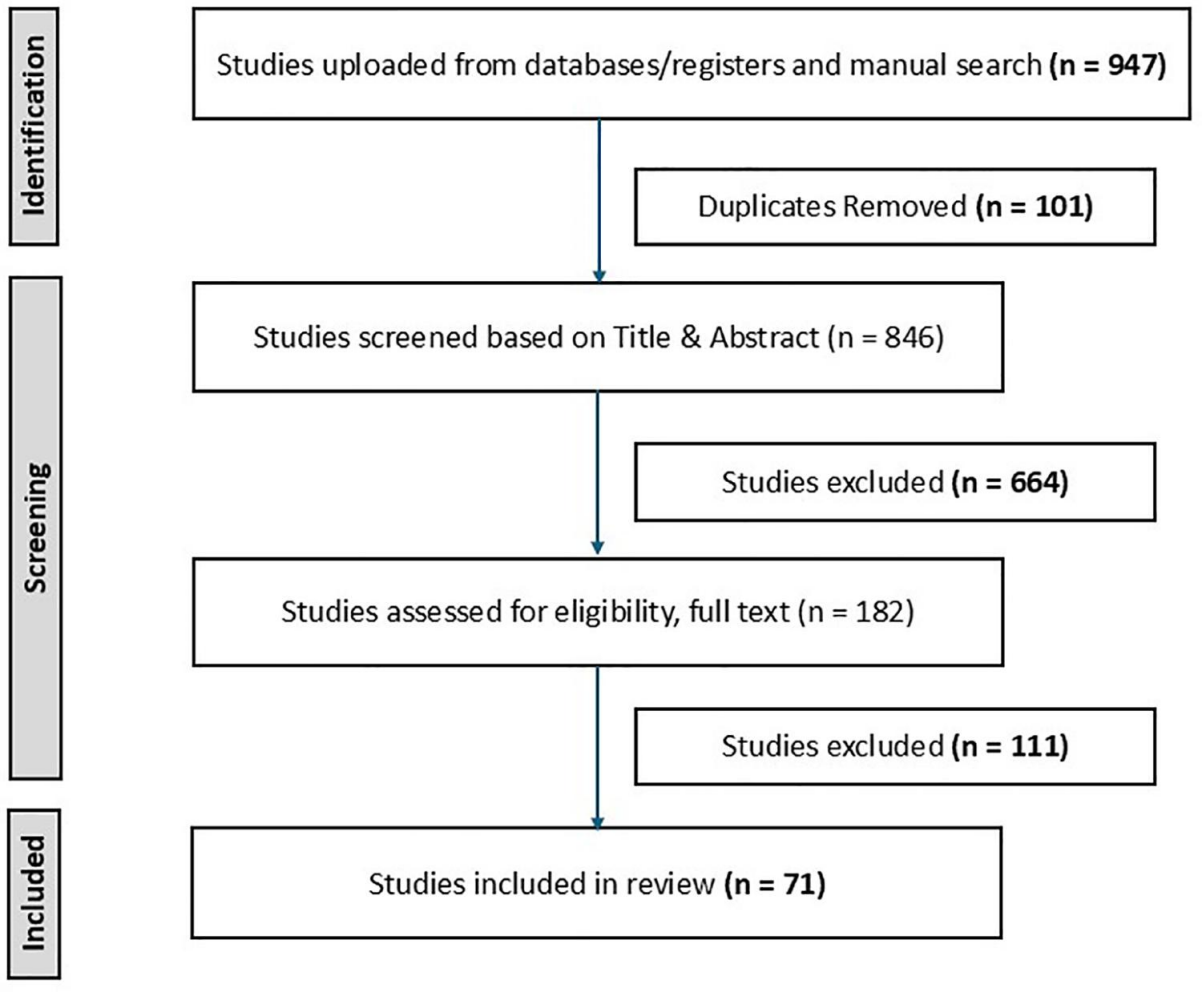
PRISMA diagram showing the search and study selection process.

The PRISMA diagram illustrates the study selection process (Figure 1). A total of 947 studies were imported for screening. After removing 101 duplicates (identified by Covidence and manual screening), 846 studies were screened based on titles and abstracts. Screening excluded 664 studies, leaving 182 articles for full-text review. Of these, 71 studies met the inclusion criteria and were included in this review of long COVID cardiovascular conditions. A summary of the characteristics of the included studies is provided in Supplementary Table 2a–c.

### 3.1 Study characteristics

#### 3.1.1 Year of publication and country

Most studies were published recently, with the highest number appearing in 2023 (*n* = 30), followed by 2022 (*n* = 27), 2021 (*n* = 8), and 2024 (*n* = 5). Only one study was published in 2020. Geographically, the majority of studies originated from Europe (45%), followed by Asia (24%), North America (17%), and South America (7%). Smaller proportions were conducted in Eurasia (3%), Africa (1%), Oceania (1%), and Europe/Asia combined (1%).

#### 3.1.2 Study design

Among the 71 included studies, observational designs predominated, accounting for 89% of all included studies. These comprised prospective cohort studies (*n* = 57), retrospective cohort studies (*n* = 14), descriptive observational studies, and case–control studies. Interventional designs accounted for 3% of all studies and consisted exclusively of randomised controlled trials (RCTs), which are not classified as observational. A total of 20 studies (28%) included control groups, comprising 9 RCTs (interventional) and 11 case–control studies (observational).

#### 3.1.3 Follow-up duration

Follow-up periods varied widely, ranging from less than 1 month to over 24 months, reflecting variability in the definitions and measurements of long COVID. For analysis, follow-up durations were categorised as short-term (<3 months; 39.4%), medium-term (3–12 months; 42.3%), long-term (>12 months; 15.5%), and not reported (2.8%).

### 3.2 Participant characteristics

#### 3.2.1 COVID testing method and vaccination status

COVID-19 diagnosis was confirmed by laboratory testing (PCR, rapid antigen test, antibody testing) in most studies (73%) (22–68). Six studies (9%) used a combination of laboratory and clinical or coded diagnoses (19, 69–73), three studies (4%) confirmed COVID-19 without specifying the method (74–76), and 10 studies (14%) did not report diagnostic methods (20, 77–85). Vaccination status was poorly reported: 52 studies (73%) did not mention vaccination status (19, 20, 22, 26–35, 37, 38, 41–63, 66, 68, 72, 74–80, 84–87), 10 studies (14%) reported mixed or partial vaccination (23, 39, 40, 64, 65, 69, 71, 82, 88, 89), six studies (8%) explicitly stated that participants were vaccinated (24, 36, 67, 70, 81, 90), and three studies (4%) reported all participants as unvaccinated (25, 73, 83).

#### 3.2.2 Comorbidity assessment

Most studies (77.5%) reported comorbidities or underlying health conditions (19, 20, 23–26, 28, 31–33, 37, 39–41, 43–55, 57–61, 63–67, 69–72, 74–77, 79–83, 85–90), while nearly one-fourth (22.5%) did not (22, 27, 29, 30, 34–36, 38, 42, 56, 62, 68, 73, 78, 84). The methods used to assess comorbidities varied considerably. Some studies focused solely on cardiovascular outcomes without providing comprehensive baseline health data. A few studies reported only body mass index, which was insufficient for a thorough assessment of comorbidities.

#### 3.2.3 Cardiovascular parameters measured and methods used

Across the 71 studies, a total of 90 cardiovascular measurements were reported, reflecting a comprehensive cardiac assessment. Common methods included were echocardiography (*n* = 40) (20, 24, 27–30, 32–36, 39–42, 46, 47, 50, 51, 54–56, 59, 63, 65, 67, 68, 75–77, 79, 84–89), cardiac biomarkers (troponin, NT-proBNP) (*n* = 10) (23, 27, 30, 32, 38, 53, 60, 65, 86, 88), cardiac magnetic resonance (CMR) imaging (*n* = 8) (27, 28, 34, 40, 54, 67, 79, 84), cardiopulmonary exercise testing (*n* = 9) (24, 30, 46, 52, 56, 59, 68, 74, 80, 87), BP/HR monitoring (*n* = 7) (25, 26, 31, 36, 45, 65, 69, 87), 24-h Holter monitoring (*n* = 3) (44, 49, 78), and coronary angiography (*n* = 2) (38, 57). Key findings included arrhythmias in 11 studies (22, 24, 26, 27, 31, 44, 45, 49, 60, 61, 78), impaired cardiac function/morphology in 38 studies (20, 24, 27–30, 32–35, 37, 39–43, 46–48, 50, 51, 54–56, 59, 63, 67–69, 75–77, 79, 84, 85, 87–89), elevated biomarkers in 11 studies (23, 27, 30, 32, 38, 48, 53, 60, 65, 86, 88), and reduced exercise capacity in 13 studies (24, 30, 31, 33, 45, 46, 52, 56, 59, 68, 74, 80, 87), highlighting the multisystem cardiovascular impact.

#### 3.2.4 Long COVID definition

Definitions or descriptors of long COVID were provided in 41 studies (58%), while 30 studies (42%) did not define the term (20, 24, 26–29, 31, 43, 44, 51, 54, 56, 60, 61, 64, 67, 68, 70, 71, 73, 76, 77, 81, 83–85, 90). Six studies (8%) applied formal definitions from international health organisations [e.g., World Health Organization (WHO), Centers for Disease Control and Prevention (CDC), NICE] (30, 36, 52, 58, 65, 79), while 15 studies (21%) used study-specific symptom- or duration-based definitions (19, 23, 25, 32–34, 37, 40–42, 46, 49, 57, 74, 78, 86). Formal definitions commonly emphasised symptoms persisting beyond the acute phase and the exclusion of alternative diagnoses.

There was no consistent definition of long COVID; however, most studies identified a post-infection timeframe and persistent symptoms that could not be explained by other causes.

#### 3.2.5 Reporting long COVID symptoms and symptom severity

Methods of symptom reporting varied, including recording the number of symptoms, describing their nature, or reporting both. Symptoms of long COVID were described in 25 studies (19, 20, 35, 38, 42, 46–50, 53, 55, 57–59, 62, 65, 74, 78, 80, 85–89). Symptoms were not reported or were poorly defined, whereas others used classification systems such as the New York Heart Association (NYHA) functional classification.

Symptom severity reporting across the reviewed studies demonstrated considerable variability. Approximately one-third of studies (31%) used broad classifications such as “mild to severe” (19, 20, 23, 29–31, 34, 35, 39, 40, 43, 45, 48, 50, 55, 56, 62, 69, 70, 74, 81, 90). A smaller proportion focused specifically on severe cases (7%) (25, 33, 42, 63, 67), while only one study (1%) described exclusively mild symptoms (42). Intermediate categories were also used, including “moderate to severe” (8%) (36, 37, 55, 70, 74), “mild to moderate” (3%) (34, 52), and “severe to mild” (1%) (42), highlighting inconsistencies in severity classification. A substantial number of studies (25%) did not clearly define symptom severity (24, 38, 41, 46, 47, 51, 57, 59, 61, 65, 66, 75, 76, 79, 80, 82, 88, 89), and nearly one-quarter (23%) did not report symptom severity at all (22, 26, 27, 32, 44, 49, 53, 54, 60, 64, 68, 71–73, 77, 78, 83–85).

#### 3.2.6 Hospitalisation status (hospitalised vs. non-hospitalised)

The findings in this section are descriptive rather than comparative, as most included studies either used mixed cohorts or did not stratify outcomes by hospitalisation status. As a result, meaningful comparisons between hospitalised and non-hospitalised groups were limited by heterogeneity in study design, inconsistent reporting of key variables—including vaccination status and baseline cardiovascular risk—and variable follow-up durations. Substantial variation in the timing and modalities of cardiovascular assessments further constrained the ability to draw direct comparisons.

Across the included studies, hospitalisation status was reported as follows: 51% enrolled only hospitalised participants (19, 22, 25, 27–34, 41, 43, 46, 48–51, 53, 57, 59–63, 70, 72, 73, 75, 76, 79, 82, 84, 87, 89), 25% included mixed hospitalised and non-hospitalised cohorts (19, 23, 30, 35, 38–40, 46–49, 52, 55, 56, 69, 77, 81, 90), 11% included only non-hospitalised individuals (20, 36, 44, 45, 54, 66, 68, 86), and 13% did not report hospitalisation status (24, 26, 37, 42, 74, 78, 80, 83, 88). In mixed-cohort studies, the proportion of hospitalised participants was often small, making it difficult to attribute outcomes solely to hospitalisation status.

Among the 25 studies reporting long COVID symptoms (19, 20, 35, 38, 42, 46–50, 53, 55, 57–59, 62, 65, 74, 78, 80, 85–89), hospitalisation status was variably documented and inconsistently linked to symptom profiles. Because methods of symptom assessment differed widely and were rarely stratified by hospitalisation status, detailed symptom outcomes are presented separately in Section 3.3.1.

Overall, differences between hospitalised and non-hospitalised groups must be interpreted with caution, as the lack of consistent stratification and considerable methodological limitations across studies precluded reliable direct comparisons.

### 3.3 Outcome characteristics—development of cardiovascular events associated with long COVID

To enhance clarity and improve clinical interpretability, cardiovascular outcomes were categorised into four predefined domains:

1. Symptoms: patient-reported cardiovascular complaints, such as chest pain, palpitations, dyspnoea, dizziness, or fatigue.
2. Imaging findings: abnormalities detected on echocardiography, CMR imaging, or other cardiac imaging modalities.
3. Biomarkers: laboratory indicators of cardiac injury or stress, including troponin and NT-proBNP.
4. Hard clinical endpoints: major adverse cardiovascular events (MACE), including myocardial infarction, heart failure, stroke, thromboembolism, and cardiac death.

Outcomes were synthesised using these *a priori* extraction domains, and each domain is presented in detail in Sections 3.3.1–3.3.4.

#### 3.3.1 Symptoms

Persistent cardiovascular symptoms—including palpitations, dizziness, fatigue, and dyspnoea—were reported across 25 studies assessing long COVID manifestations (19, 20, 35, 42, 46–50, 53, 55, 57–59, 62, 65, 74, 78, 80, 85–89). Dysautonomia syndromes, including orthostatic hypotension and postural tachycardia syndrome, were also observed; one study reported a prevalence of 15.21% among individuals with long COVID (45). Longitudinal evidence indicated that symptoms may persist or newly emerge months after infection, with a more pronounced symptom burden observed in individuals who experienced more severe acute illness (20, 27, 32, 33, 75).

#### 3.3.2 Imaging findings

Cardiac imaging abnormalities were identified across a range of studies, regardless of acute hospitalisation status. Multiple investigations reported arrhythmias and ventricular dysfunction associated with long COVID (22, 24, 26, 27, 31, 44, 45, 49, 60, 61, 78). Echocardiographic abnormalities were documented in up to 70% of patients within the first 3 months after infection, particularly among those with moderate to severe acute disease (55). CMR studies demonstrated evidence of subclinical myocardial injury in both hospitalised individuals and up to 8% of non-hospitalised patients (20, 27–29, 34, 40, 54, 67, 79, 84). Measures such as ventricular strain and diastolic function remained abnormal in several longitudinal studies (27, 55, 63, 75).

#### 3.3.3 Biomarkers

Elevations in cardiac biomarkers—including troponin and NT-proBNP—were reported in 11 studies, suggesting persistent myocardial injury or cardiac stress following COVID-19 infection (23, 27, 30, 32, 38, 48, 53, 60, 65, 86, 88). These abnormalities frequently corresponded with structural or functional changes observed on imaging, reinforcing the presence of subclinical myocardial injury. Persistent biomarker elevation was also noted in individuals reporting ongoing cardiac dysfunction or dysautonomia. Vaccination was associated with a lower likelihood of biomarker-defined cardiac injury (89).

#### 3.3.4 Hard clinical endpoints (MACE and other events)

Several studies identified an increased risk of MACE—including heart failure, myocardial infarction, stroke, and thromboembolism—among individuals with prior COVID-19 compared with controls (64, 83, 90). Vulnerability appeared to be higher among older adults and individuals with pre-existing cardiovascular risk factors (71). Vaccination was associated with a reduced risk of MACE (89).

## 4 Discussion

This narrative synthesis review demonstrates that COVID-19 is associated with persistent cardiovascular complications across all levels of disease severity. Hospitalised patients, particularly those requiring intensive care, are at the highest risk of adverse outcomes, including MACE, myocardial injury, heart failure, arrhythmias, and structural or functional cardiac abnormalities, with imaging frequently demonstrating myocardial fibrosis and ventricular dysfunction. Although less pronounced, subclinical myocardial dysfunction, autonomic disturbances, and subtle arrhythmias were also observed in non-hospitalised patients following mild infection. Vaccination was associated with reduced rates of persistent cardiac injury and symptoms, suggesting a protective effect against long-term cardiovascular sequelae.

Our findings are consistent with previous literature reporting persistent cardiovascular manifestations following COVID-19 infection. Prior scoping and systematic reviews have documented a broad range of long COVID cardiovascular outcomes, including myocardial injury, arrhythmias, heart failure, thromboembolic events, and structural abnormalities (91, 92). Similarly, several systematic reviews and meta-analyses have reported increased long-term risks of cardiovascular complications, particularly among older individuals and those with pre-existing cardiovascular risk factors (13, 93). These findings align with the present review and underscore both the persistence of cardiovascular sequelae across disease severities—most pronounced among hospitalised populations—and the considerable heterogeneity in reported prevalence.

Arrhythmias have been a prominent focus of earlier research. Previous studies reported a high prevalence of arrhythmias, particularly atrial fibrillation, in patients with COVID-19, with proposed mechanisms including inflammation, hypoxia, myocardial injury, and electrolyte imbalance (94, 95). Consistent with this literature, arrhythmias were among the most frequently reported cardiovascular findings in long COVID in this review, particularly in hospitalised patients. However, ongoing rhythm disturbances were also identified in non-hospitalised individuals.

Cardiovascular involvement has also been increasingly recognised in non-hospitalised populations. Studies have reported subclinical myocardial dysfunction, dysautonomia, and persistent cardiac symptoms, such as palpitations, chest pain, and dyspnoea, occurring with or without objective evidence of cardiovascular disease (8, 10). In the present review, non-hospitalised individuals similarly demonstrated subclinical myocardial and autonomic abnormalities, reinforcing the need for clinical vigilance beyond hospitalised cohorts.

Symptom profiles varied according to hospitalisation status. Cardiovascular symptoms were more frequently reported among hospitalised patients, whereas non-hospitalised populations more commonly reported fatigue and dyspnoea, with less prominent cardiovascular symptoms. Fatigue may partially reflect cardiovascular involvement, as right ventricular dysfunction and increased pulmonary artery pressures have been documented in some patients during recovery (96).

Across the included studies, multiple cardiovascular domains were assessed, including arrhythmias, haemodynamic parameters, cardiac function and structure, biomarkers, exercise capacity, and autonomic function; this explains why the number of reported measurements exceeded the number of studies. Echocardiographic assessment identified a substantial burden of cardiac dysfunction across all patient groups, with a higher prevalence among hospitalised cohorts. Findings included left ventricular dysfunction, reduced ejection fraction, and impaired global longitudinal strain, reported irrespective of hospitalisation status.

In this review, vaccinated individuals exhibited lower rates of persistent cardiac injury and symptoms. Consistent with these findings, Verdecchia et al. reported that although rare cardiovascular adverse events, such as myocarditis, have been observed following COVID-19 vaccination particularly among younger males the overall cardiovascular benefits of vaccination substantially outweigh these risks (97). Paknahad et al. further demonstrated that cardiovascular adverse events vary by vaccine platform but remain uncommon relative to the cardiovascular risks associated with SARS-CoV-2 infection (98). Overall, these findings clearly establish vaccination as a critical intervention for reducing both acute COVID-19 severity and long-term cardiovascular sequelae. The cardiovascular risks associated with SARS-CoV-2 infection overwhelmingly exceed those linked to vaccination, reinforcing strong and ongoing recommendations for vaccination to prevent severe disease and subsequent cardiac complications.

Comparisons between hospitalised and non-hospitalised groups in this review were constrained by heterogeneity in study design, inconsistent reporting of key variables including vaccination status and baseline cardiovascular risk and variable follow-up durations. Furthermore, substantial variation in the timing and modalities of cardiovascular assessments limited direct comparisons and complicated the interpretation of disease progression. These methodological limitations highlight the need for standardised, time-based definitions and assessment protocols in long COVID research.

Long COVID definitions vary substantially across studies, resulting in inconsistent prevalence estimates and limiting comparability (99, 100). The absence of a standardised, criterion-based definition further complicates clinical assessment and research. Differences in symptom duration, symptom types, and requirements for confirmed SARS-CoV-2 infection contribute to this variability. The most commonly used definitions are alternative definitions, followed by those proposed by the WHO, NICE, and CDC. These inconsistencies directly influence prevalence rates and impede accurate diagnosis, targeted treatment, and effective identification of affected individuals (99, 100). In this review, definitions of long COVID varied widely across the included studies, with many relying solely on symptom persistence without explicit diagnostic criteria. This lack of standardisation impairs comparability and limits the interpretation of cardiovascular outcomes. Future research should prioritise harmonised definitions and consistent reporting of key clinical variables to improve understanding of long-term cardiovascular sequelae following COVID-19.

### 4.1 Limitations

The aim of this review was to provide a narrative synthesis of studies aligned with the research objectives. Although a standardised search strategy was used, some relevant articles may have been missed. We also acknowledge the potential for selection bias inherent in narrative reviews. To minimise this risk, inclusion and exclusion criteria were predefined, and study screening and data extraction were performed independently by two authors.

A key limitation of this review is that many included studies enrolled mixed or clinically heterogeneous populations and did not report stratified outcomes. Moreover, the absence of a standardised, criterion-based long COVID definition undermines the comparability of findings across studies. The lack of stratified data and a unified definition limits the assessment of differences between clinically relevant groups and reduces the precision of the conclusions. As a result, the synthesis relies on aggregated findings, which further limits the precision and generalisability of the conclusions. Future research should include predefined subgroup analyses and report them transparently to improve the interpretability and applicability of findings.

### 4.2 Future study

Overall, the evidence supports an increased cardiovascular risk and the need for ongoing monitoring in hospitalised COVID-19 patients, while recognising that non-hospitalised individuals may also require surveillance for subtle cardiac changes*.* Future prospective research using standardised methods is crucial to clarify these risks and inform tailored follow-up care. In addition, future research should incorporate predefined subgroup analyses, report them transparently, and adopt a standardised definition of long COVID to support consistent clinical practice, improve research comparability, and inform policy.

## 5 Conclusion

COVID-19 can lead to persistent cardiovascular complications in both hospitalised and non-hospitalised patients. Hospitalised patients are at the highest risk of major cardiovascular events and structural abnormalities, whereas non-hospitalised patients may experience subclinical myocardial dysfunction and autonomic disturbances. Vaccination may mitigate long-term cardiac sequelae, highlighting its protective role beyond the acute phase of the illness. These findings emphasise the importance of ongoing cardiovascular surveillance for all COVID-19 survivors*.* Future prospective studies using standardised methods are needed to clarify risk profiles and inform tailored long COVID cardiovascular care.

## Data Availability

The original contributions presented in the study are included in the article/Supplementary Material, further inquiries can be directed to the corresponding author.
