# Supplementary material for: Long-term cardiovascular impact of COVID-19 among hospitalised and non-hospitalised populations: a narrative synthesis review
Source: Front Cardiovasc Med. 2026 May 7;13:1741293. doi: 10.3389/fcvm.2026.1741293 (PMC13190603; doi:10.3389/fcvm.2026.1741293)
Supplement: Supplementary file 1 [file Table1.docx]

| PubMed | ("Post-Acute COVID-19 Syndrome"[MeSH Terms] OR "long COVID"[Title/Abstract: ~2] OR "long COVID syndrome"[All Fields] OR "long COVID-19 syndrome"[All Fields]) AND "Cardiovascular Diseases"[MeSH Terms] |
| --- | --- |
| Embase | ((covid, long-haul or covid-19, long haul or covid-19 post-acute sequelae or covid-19 syndrome, post-acute or long covid or long haul covid or long haul covid 19 or long haul covid-19s or long-haul covids or "pasc post-acute sequelae of covid 19" or post-acute covid 19 syndrome or "post-acute sequelae of covid 19" or "post-acute sequelae of sars cov 2 infection" or post covid conditions or post-acute covid-19 syndromes or post-covid condition) and (cardiac disease or cardiac diseases or cardiac disorder or cardiac disorders or heart disease or heart diseases or heart disorder or heart disorders)).mp. |
| Medline | ((covid, long-haul or covid-19, long haul or covid-19 post-acute sequelae or covid-19 syndrome, post-acute or long covid or long haul covid or long haul covid 19 or long haul covid-19s or long-haul covids or "pasc post-acute sequelae of covid 19" or post-acute covid 19 syndrome or "post-acute sequelae of covid 19" or "post-acute sequelae of sars cov 2 infection" or post covid conditions or post-acute covid-19 syndromes or post-covid condition) and (cardiac disease or cardiac diseases or cardiac disorder or cardiac disorders or heart disease or heart diseases or heart disorder or heart disorders)).mp. |
| CINAHL Complete | ((MH "Cardiovascular Diseases+") OR "cardiovascular disease") AND ((MH "Post-Acute COVID-19 Syndrome") OR "long covid") |

**Supplementary Table 1: Search Strategy**
