# Supplementary material for: Long-term cardiovascular impact of COVID-19 among hospitalised and non-hospitalised populations: a narrative synthesis review
Source: Front Cardiovasc Med. 2026 May 7;13:1741293. doi: 10.3389/fcvm.2026.1741293 (PMC13190603; doi:10.3389/fcvm.2026.1741293)
Supplement: Supplementary file 2 [file Datasheet1.zip › Supplementary Table 2a.docx]

**Supplementary Table 2a: Characteristics of included studies (Study Characteristics)**

| **Reference** | **Year of publication** | **Country** | **Study Design** | **Long Covid Definition** | **Duration of follow up post COVID** |
| --- | --- | --- | --- | --- | --- |
| 22 | 2023 | Australia | Descriptive | Symptoms Only | Short-term (<3 months) |
| 23 | 2024 | USA | Analytic - Observational | Study-Specific Time-Based | Long-term (>12 months) |
| 77 | 2023 | Argentina | Analytic - Observational | Not Defined | Short-term (<3 months) |
| 78 | 2023 | China | Analytic - Observational | Study-Specific Time-Based | Short-term (<3 months) |
| 24 | 2022 | Israel | Analytic - Observational | Not Defined | Medium-term (3–12 months) |
| 25 | 2023 | UK | Analytic - Observational | Study-Specific Time-Based | Long-term (>12 months) |
| 26 | 2023 | Brazil | Analytic - Observational | Not Defined | Medium-term (3–12 months) |
| 27 | 2022 | Norway | Analytic - Observational | Not Defined | Short-term (<3 months) |
| 28 | 2020 | China | Analytic - Observational | Not Defined | Short-term (<3 months) |
| 29 | 2022 | Poland | Analytic - Observational | Not Defined | Short-term (<3 months) |
| 30 | 2022 | Denmark | Analytic - Observational | Guideline-Based | Medium-term (3–12 months) |
| 31 | 2023 | Poland | Analytic - Observational | Not Defined | Medium-term (3–12 months) |
| 32 | 2023 | Austria | Analytic - Observational | Study-Specific Time-Based | Medium-term (3–12 months) |
| 33 | 2022 | Germany | Analytic - Observational | Study-Specific Time-Based | Medium-term (3–12 months) |
| 34 | 2021 | Romania | Analytic - Observational | Study-Specific Time-Based | Short-term (<3 months) |
| 35 | 2021 | USA | Analytic - Observational | Symptoms Only | Short-term (<3 months) |
| 36 | 2022 | United Kingdom | Analytic - Observational | Guideline-Based | Medium-term (3–12 months) |
| 79 | 2022 | Japan | Analytic - Observational | Guideline-Based | Medium-term (3–12 months) |
| 37 | 2023 | India | Analytic - Interventional | Study-Specific Time-Based | Medium-term (3–12 months) |
| 69 | 2022 | USA | Analytic - Observational | Symptoms Only | Medium-term (3–12 months) |
| 38 | 2021 | Germany | Analytic - Observational | Symptoms Only | Medium-term (3–12 months) |
| 39 | 2023 | India | Analytic - Observational | Symptoms Only | Medium-term (3–12 months) |
| 40 | 2022 | Germany | Analytic - Observational | Study-Specific Time-Based | Short-term (<3 months) |
| 80 | 2023 | USA | Descriptive | Symptoms Only | Short-term (<3 months) |
| 81 | 2023 | USA | Analytic - Observational | Not Defined | Short-term (<3 months) |
| 41 | 2022 | Morocco | Descriptive | Study-Specific Time-Based | Short-term (<3 months) |
| 42 | 2022 | Romania | Analytic - Observational | Study-Specific Time-Based | NR |
| 43 | 2022 | Israel | Analytic - Observational | Not Defined | Short-term (<3 months) |
| 82 | 2024 | China | Analytic - Observational | Symptoms Only | NR |
| 44 | 2022 | Turkey | Analytic - Observational | Not Defined | Short-term (<3 months) |
| 45 | 2022 | India | Analytic - Observational | Symptoms Only | Short-term (<3 months) |
| 46 | 2024 | Italy | Analytic - Observational | Study-Specific Time-Based | Medium-term (3–12 months) |
| 74 | 2024 | Brazil | Analytic - Observational | Study-Specific Time-Based | Medium-term (3–12 months) |
| 20 | 2022 | Germany | Analytic - Observational | Not Defined | Long-term (>12 months) |
| 47 | 2021 | Italy | Analytic - Observational | Symptoms Only | Medium-term (3–12 months) |
| 83 | 2022 | Taiwan | Descriptive | Not Defined | Long-term (>12 months) |
| 70 | 2023 | Iran | Analytic - Observational | Not Defined | Medium-term (3–12 months) |
| 84 | 2023 | Czech Republic | Analytic - Observational | Not Defined | Short-term (<3 months) |
| 19 | 2021 | England | Analytic - Observational | Study-Specific Time-Based | Medium-term (3–12 months) |
| 88 | 2024 | USA | Analytic - Observational | Symptoms Only | Medium-term (3–12 months) |
| 48 | 2023 | Argentina | Analytic - Observational | Symptoms Only | Short-term (<3 months) |
| 71 | 2023 | Hong Kong and UK | Analytic - Observational | Not Defined | Long-term (>12 months) |
| 49 | 2022 | Brazil | Analytic - Observational | Study-Specific Time-Based | Short-term (<3 months) |
| 50 | 2022 | India | Analytic - Observational | Symptoms Only | Medium-term (3–12 months) |
| 85 | 2022 | USA | Analytic - Observational | Symptoms Only | Short-term (<3 months) |
| 51 | 2023 | Ukrane | Analytic - Observational | Not Defined | Short-term (<3 months) |
| 52 | 2023 | Isarael | Analytic - Interventional | Guideline-Based | Medium-term (3–12 months) |
| 53 | 2023 | Turkey | Analytic - Observational | Symptoms Only | Long-term (>12 months) |
| 54 | 2023 | Poland | Analytic - Observational | Not Defined | Medium-term (3–12 months) |
| 55 | 2023 | Mexico | Analytic - Observational | Symptoms Only | Short-term (<3 months) |
| 56 | 2022 | Spain | Analytic - Observational | Not Defined | Short-term (<3 months) |
| 89 | 2023 | Argentina | Analytic - Observational | Symptoms Only | Short-term (<3 months) |
| 90 | 2022 | USA | Analytic - Observational | Not Defined | Medium-term (3–12 months) |
| 57 | 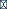2024 | China | Analytic - Observational | Study-Specific Time-Based | Short-term (<3 months) |
| 86 | 2022 | Norway | Descriptive | Study-Specific Time-Based | Long-term (>12 months) |
| 58 | 2022 | United States | Analytic - Observational | Guideline-Based | Long-term (>12 months) |
| 59 | 2023 | Ukraine | Analytic - Observational | Symptoms Only | Short-term (<3 months) |
| 60 | 2021 | China | Analytic - Observational | Not Defined | Medium-term (3–12 months) |
| 61 | 2021 | Italy | Analytic - Observational | Not Defined | Short-term (<3 months) |
| 62 | 2023 | Italy | Analytic - Observational | Symptoms Only | Medium-term (3–12 months) |
| 75 | 2023 | Italy | Analytic - Observational | Symptoms Only | Long-term (>12 months) |
| 63 | 2022 | Netherlands | Analytic - Observational | Symptoms Only | Short-term (<3 months) |
| 64 | 2024 | Canada | Analytic - Observational | Not Defined | Long-term (>12 months) |
| 65 | 2024 | Poland | Analytic - Observational | Guideline-Based | Long-term (>12 months) |
| 76 | 2021 | Denmark | Analytic - Observational | Not Defined | Short-term (<3 months) |
| 66 | 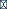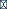2021 | Germany | Analytic - Observational | Symptoms Only | Medium-term (3–12 months) |
| 67 | 2022 | Russia | Descriptive | Not Defined | Medium-term (3–12 months) |
| 87 | 2023 | Poland | Analytic - Observational | Symptoms Only | Medium-term (3–12 months) |
| 72 | 2021 | United Kingdom | Analytic - Observational | Not Defined | Medium-term (3–12 months) |
| 73 | 2023 | USA | Analytic - Observational | Not Defined | Medium-term (3–12 months) |
| 68 | 2023 | Germany | Analytic - Observational | Not Defined | Medium-term (3–12 months) |
