# Supplementary material for: Long-term cardiovascular impact of COVID-19 among hospitalised and non-hospitalised populations: a narrative synthesis review
Source: Front Cardiovasc Med. 2026 May 7;13:1741293. doi: 10.3389/fcvm.2026.1741293 (PMC13190603; doi:10.3389/fcvm.2026.1741293)
Supplement: Supplementary file 2 [file Datasheet1.zip › Supplementary Table 2b.docx]

**Supplementary Table 2b: Characteristics of included studies (Participant Characteristics)**

| **Reference** | **COVID 19 testing method** | **Comorbidities** | **Cardiovascular parameters** | **Methods used to measure cardiovascular parameters** | **Vaccination status** | **Symptom severity** | **Hospital Status** |
| --- | --- | --- | --- | --- | --- | --- | --- |
| 22 | Lab-confirmed (PCR, RAT, antibody) | No | Arrhythmias & Heart Rhythms | ECG, 24-h Holter monitoring, ambulatory ECG | NR | NR | Only Hospitalised |
| 23 | Lab-confirmed (PCR, RAT, antibody) | Yes | BP & HR, Ischemic and Coronary Disease Indicators, Symptoms & Outcomes, Cardiac Biomarkers | BP monitoring, ABPM, ECG, clinical exam; ECG, CMR, coronary angiography, PET; clinical assessments, lab biomarkers; blood tests | Mixed / Partially reported | Severe | Mixed |
| 77 | NR | Yes | Cardiac Function & Physiology | Echocardiography (TTE), CMR, Doppler, STE | NR | NR | Mixed |
| 78 | NR | No | Arrhythmias & Heart Rhythms, HRV & Autonomic Function | ECG, 24-h Holter monitoring, Polar HR monitors | NR | NR | NR |
| 24 | Lab-confirmed (PCR, RAT, antibody) | Yes | Arrhythmias & Heart Rhythms, Cardiac Function & Physiology, Symptoms & Clinical Outcomes, Exercise Capacity & Functional Status | ECG, TTE, CPET, 6MWT; clinical assessments, lab biomarkers | Vaccinated | Not clearly defined | NR |
| 25 | Lab-confirmed (PCR, RAT, antibody) | Yes | BP & HR, Ischemic and Coronary Disease Indicators, Symptoms & Clinical Outcomes | BP monitoring, ECG, clinical exam; coronary angiography; clinical assessments | Not vaccinated | Severe (critical care) | Only Hospitalised |
| 26 | Lab-confirmed (PCR, RAT, antibody) | Yes | Arrhythmias & Heart Rhythms, BP & HR | ECG, 24-h Holter monitoring | NR | NR | NR |
| 27 | Lab-confirmed (PCR, RAT, antibody) | No | Arrhythmias & Heart Rhythms, Cardiac Function & Physiology, Cardiac Morphology & Structure, Cardiac Biomarkers | ECG, TTE, CMR, Doppler; blood tests | NR | NR | Only Hospitalised |
| 28 | Lab-confirmed (PCR, RAT, antibody) | Yes | Cardiac Function & Physiology, Cardiac Morphology & Structure | CMR | NR | Moderate to severe | Only Hospitalised |
| 29 | Lab-confirmed (PCR, RAT, antibody) | No | Cardiac Function & Physiology | CMR, ECG | NR | Moderate to severe | Only Hospitalised |
| 30 | Lab-confirmed (PCR, RAT, antibody) | No | Cardiac Function & Physiology, Symptoms & Clinical Outcomes, Cardiac Biomarkers, Exercise Capacity & Functional Status | Echocardiography (TTE), CMR, 6MWT, CPET, lab biomarkers | NR | Mild to severe | Mixed |
| 31 | Lab-confirmed (PCR, RAT, antibody) | Yes | Arrhythmias & Heart Rhythms, BP & HR, Symptoms & Clinical Outcomes, Exercise Capacity & Functional Status | ECG, 24-h Holter monitoring, BP, clinical assessments | NR | Mild to severe | Only Hospitalised |
| 32 | Lab-confirmed (PCR, RAT, antibody) | Yes | Cardiac Function & Physiology, Symptoms & Clinical Outcomes, Cardiac Biomarkers | TTE, CMR, clinical assessments, blood tests | NR | NR | Only Hospitalised |
| 33 | Lab-confirmed (PCR, RAT, antibody) | Yes | Cardiac Function & Physiology, Exercise Capacity & Functional Status | Echocardiography, CPET, 6MWT | NR | Severe | Only Hospitalised |
| 34 | Lab-confirmed (PCR, RAT, antibody) | No | Cardiac Function & Physiology, Cardiac Morphology & Structure | Echocardiography (TTE, Doppler) | NR | Mild to moderate | Only Hospitalised |
| 35 | Lab-confirmed (PCR, RAT, antibody) | No | Cardiac Function & Physiology | ECG, CMR | NR | Mild to severe | Mixed |
| 36 | Lab-confirmed (PCR, RAT, antibody) | No | BP & HR , Cardiac Function & Physiology | BP, Echocardiography, CMR | Vaccinated | Moderate to severe | Not Hospitalised |
| 79 | NR | Yes | Cardiac Morphology & Structure, Cardiac Function & Physiology | Echocardiography, ECG, CMR, SPECT/CT | NR | Not clearly defined | Only Hospitalised |
| 37 | Lab-confirmed (PCR, RAT, antibody) | Yes | Cardiac Function & Physiology | Echocardiography, ECG | NR | Moderate to severe | NR |
| 69 | Mixed (lab-confirmed + clinical/ICD) | Yes | BP & HR , Cardiac Function & Physiology | BP, ECG, Holter monitoring, TTE, Cardiac MRI | Mixed / Partially | Mild to severe | Mixed |
| 38 | Lab-confirmed (PCR, RAT, antibody) | No | Ischemic and Coronary Disease Indicators, Cardiac Biomarkers, Cardiac Function & Physiology | ECG, CMR, lab biomarkers | NR | Not clearly defined | Mixed |
| 39 | Lab-confirmed (PCR, RAT, antibody) | Yes | Cardiac Function & Physiology | Echocardiography, Speckle-tracking echocardiography | Mixed / Partially | Mild to severe | Mixed |
| 40 | Lab-confirmed (PCR, RAT, antibody) | Yes | Cardiac Function & Physiology, Cardiac Morphology & Structure | CMR imaging, ECG, echocardiography | Mixed / Partially | Mild to severe | Mixed |
| 80 | NR | Yes | Ischemic and Coronary Disease Indicators, Exercise Capacity & Functional Status | Coronary angiography, PET, CPET | NR | Not clearly defined | NR |
| 81 | NR | Yes | Symptoms & Clinical Outcomes | Clinical assessments | Vaccinated | Mild to severe | Mixed |
| 41 | Lab-confirmed (PCR, RAT, antibody) | Yes | Cardiac Function & Physiology, Cardiac Morphology & Structure | ECG, Echocardiography | NR | Not clearly defined | Only Hospitalised |
| 42 | Lab-confirmed (PCR, RAT, antibody) | No | Cardiac Function & Physiology, Cardiac Morphology & Structure | Echocardiography (TTE) | NR | Severe to mild | NR |
| 43 | Lab-confirmed (PCR, RAT, antibody) | Yes | Cardiac Function & Physiology | Echocardiography, Speckle tracking echocardiography | NR | Mild to severe | Only Hospitalised |
| 82 | NR | Yes | Symptoms & Clinical Outcomes | Survey | Mixed / Partially | Not clearly defined | Only Hospitalised |
| 44 | Lab-confirmed (PCR, RAT, antibody) | Yes | Arrhythmias & Heart Rhythms, HR Variability (HRV) & Autonomic Function | 24-h Holter monitoring, ECG | NR | NR | Not Hospitalised |
| 45 | Lab-confirmed (PCR, RAT, antibody) | Yes | Arrhythmias & Heart Rhythms, BP & HR, Exercise Capacity & Functional Status | ECG, BP, exercise tolerance tests | NR | Mild to severe | NR |
| 46 | Lab-confirmed (PCR, RAT, antibody) | Yes | Cardiac Function & Physiology, Exercise Capacity & Functional Status | ECG, Holter, TTE, cardiopulmonary exercise testing | NR | Not clearly defined | Mixed |
| 74 | Confirmed COVID-19 but method did not specify | Yes | Exercise Capacity & Functional Status | Cardiopulmonary testing | NR | Moderate to severe | NR |
| 20 | NR | Yes | Cardiac Function & Physiology | CMR imaging, lab biomarkers | NR | Mild to severe | Not Hospitalised |
| 47 | Lab-confirmed (PCR, RAT, antibody) | Yes | Cardiac Function & Physiology | Echocardiography, ECG | NR | Not clearly defined | Mixed |
| 83 | NR | Yes | Ischemic and Coronary Disease Indicators, Symptoms & Clinical Outcomes | Medical records, ICD codes | Not vaccinated | NR | NR |
| 70 | Mixed (lab-confirmed + clinical/ICD) | Yes | Symptoms & Clinical Outcomes | Incidence tracking | Vaccinated | Moderate to severe | Only Hospitalised |
| 84 | NR | No | Cardiac Function & Physiology, Cardiac Morphology & Structure | CMR | NR | NR | Only Hospitalised |
| 19 | Mixed (lab-confirmed + clinical/ICD) | Yes | Cardiac Morphology & Structure | MRI | NR | Moderate to severe | Mixed |
| 88 | NR | Yes | Cardiac Function & Physiology, Ischemic and Coronary Disease Indicators, Cardiac Biomarkers | Imaging, pathology, blood tests | Mixed / Partially | Not clearly defined | NR |
| 48 | Lab-confirmed (PCR, RAT, antibody) | Yes | Symptoms & Clinical Outcomes, Cardiac Function & Physiology, Cardiac Biomarkers | Clinical interviews, EKG, echocardiography, lab tests | NR | Mild to severe | Mixed |
| 71 | Mixed (lab-confirmed + clinical/ICD) | Yes | Symptoms & Clinical Outcomes | Incidence rates and hazard ratios | Mixed / Partially | NR | NR |
| 49 | Lab-confirmed (PCR, RAT, antibody) | Yes | Arrhythmias & Heart Rhythms, HR Variability (HRV) & Autonomic Function | ECG, Polar HR monitors | NR | NR | Mixed |
| 50 | Lab-confirmed (PCR, RAT, antibody) | Yes | Cardiac Function & Physiology | Transthoracic echocardiography | NR | Mild to severe | Only Hospitalised |
| 85 | NR | Yes | Cardiac Function & Physiology | Transthoracic echocardiography | NR | NR | NR |
| 51 | Lab-confirmed (PCR, RAT, antibody) | Yes | Cardiac Function & Physiology | Transthoracic echocardiography | NR | Not clearly defined | Only Hospitalised |
| 52 | Lab-confirmed (PCR, RAT, antibody) | Yes | Exercise Capacity & Functional Status | Exercise testing | NR | Mild to moderate | Mixed |
| 53 | Lab-confirmed (PCR, RAT, antibody) | Yes | Ischemic and Coronary Disease Indicators, Cardiac Biomarkers | ECG, SPECT MPI, lab pathology | NR | NR | Only Hospitalised |
| 54 | Lab-confirmed (PCR, RAT, antibody) | Yes | Cardiac Function & Physiology, Cardiac Morphology & Structure | Echocardiography, cardiac MRI, Holter monitoring | NR | NR | Not Hospitalised |
| 55 | Lab-confirmed (PCR, RAT, antibody) | Yes | Cardiac Function & Physiology | Transthoracic echocardiography | NR | Moderate to severe | Mixed |
| 56 | Lab-confirmed (PCR, RAT, antibody) | No | Cardiac Function & Physiology, Exercise Capacity & Functional Status | Echocardiography, Holter monitoring, 6MWT | NR | Mild to severe | Mixed |
| 89 | NR | Yes | Cardiac Function & Physiology | Echocardiography | Mixed / Partially | Not clearly defined | Only Hospitalised |
| 90 | NR | Yes | Symptoms & Clinical Outcomes | National healthcare databases | Vaccinated | Moderate to severe | Mixed |
| 57 | Lab-confirmed (PCR, RAT, antibody) | Yes | Cardiac Morphology & Structure, Ischemic and Coronary Disease Indicators | Coronary angiography, echocardiographic data assessment | NR | Not clearly defined | Only Hospitalised |
| 86 | NR | Yes | Cardiac Function & Physiology, Cardiac Biomarkers | ECG, Echocardiography, lab biomarkers | NR | Mild | Not Hospitalised |
| 58 | Lab-confirmed (PCR, RAT, antibody) | Yes | Ischemic and Coronary Disease Indicators | PET/CT myocardial blood flow and hemodynamics | NR | Not clearly defined | NR |
| 59 | Lab-confirmed (PCR, RAT, antibody) | Yes | Exercise Capacity & Functional Status, Cardiac Function & Physiology | Echocardiography, 6MWT, HR during exercise | NR | Not clearly defined | Only Hospitalised |
| 60 | Lab-confirmed (PCR, RAT, antibody) | Yes | Arrhythmias & Heart Rhythms, Cardiac Biomarkers | ECG, CMR, blood tests | NR | NR | Only Hospitalised |
| 61 | Lab-confirmed (PCR, RAT, antibody) | Yes | Arrhythmias & Heart Rhythms | ECG | NR | Not clearly defined | Only Hospitalised |
| 62 | Lab-confirmed (PCR, RAT, antibody) | No | Symptoms & Clinical Outcomes | Risk scores | NR | Mild to severe | Only Hospitalised |
| 75 | Confirmed COVID-19 but method did not specify | Yes | Cardiac Function & Physiology, Symptoms & Clinical Outcomes | Echocardiography, ECG, surveys, pathology | NR | Not clearly defined | Only Hospitalised |
| 63 | Lab-confirmed (PCR, RAT, antibody) | Yes | Cardiac Function & Physiology | Echocardiography | NR | Severe | Only Hospitalised |
| 64 | Lab-confirmed (PCR, RAT, antibody) | Yes | Symptoms & Clinical Outcomes | MACE tracking | Mixed / Partially | NR | NR |
| 65 | Lab-confirmed (PCR, RAT, antibody) | Yes | Cardiac Function & Physiology, BP & HR, Cardiac Biomarkers | Echocardiography, 24-h ECG monitoring, BP monitoring, blood tests | Mixed / Partially | Not clearly defined | NR |
| 76 | Confirmed COVID-19 but method did not specify | Yes | Cardiac Function & Physiology | Echocardiography, Speckle tracking | NR | Not clearly defined | Only Hospitalised |
| 66 | Lab-confirmed (PCR, RAT, antibody) | Yes | Cardiac Morphology & Structure | CMR-based coronary sinus flow measurements | NR | Not clearly defined | Not Hospitalised |
| 67 | Lab-confirmed (PCR, RAT, antibody) | Yes | Cardiac Function & Physiology, Cardiac Morphology & Structure | Echocardiography, MRI, 24-h ECG monitoring, coronary angiography | Vaccinated | Severe | NR |
| 87 | NR | Yes | Cardiac Function & Physiology, BP & HR , Exercise Capacity & Functional Status | Echocardiography, CPET, ECG, BP monitoring | NR | NR | Only Hospitalised |
| 72 | Mixed (lab-confirmed + clinical/ICD) | Yes | Symptoms & Clinical Outcomes | Disease incidence tracking | NR | NR | Only Hospitalised |
| 73 | Mixed (lab-confirmed + clinical/ICD) | Yes | Symptoms & Clinical Outcomes | Mortality and MACE risk stratification | Not vaccinated | NR | Only Hospitalised |
| 68 | Lab-confirmed (PCR, RAT, antibody) | No | Cardiac Function & Physiology, Exercise Capacity & Functional Status | Echocardiography, ECG, CPET | NR | NR | Not Hospitalised |

**6MWT**: six minute walk test ,**ABPM**: ambulatory blood pressure monitoring ,**ACS:** acute coronary syndrome ,**aHR:** adjusted hazard ratio**, AMR:** absolute microvascular resistance ,**ASCVD risk**: Atherosclerotic Cardiovascular Disease Risk, **BP**: Blood pressure, **CHD:** Coronary heart disease ,**CMD:** Coronary microvascular dysfunction , **CMR:** Cardiac Magnetic Resonance, **CMR:** Cardiac magnetic resonance ,**COMPASS-31:** Composite Autonomic Symptom Score (31-item questionnaire),**COVID-19**: Coronavirus disease 2019,**CPET:** Cardiopulmonary exercise test ,**CRP:** C-reactive protein ,**CV:** Cardiovascular ,**CVD**: Cardiovascular disease,**CVE:** Cardiovascular event ,**ECG:** Electrocardiogram ,**ECV:** Extracellular volume ,**EDV**: End-diastolic volume ,**EF:** ejection fraction ,**EQ-5D-5L**: EuroQol 5-Dimensions 5-Level (health-related quality of life),**GAD-7**: Generalized Anxiety Disorder-7 (anxiety),**GLS:** Global longitudinal strain ,**GW:** Global work ,**GWE:** Global work efficiency ,**GWW:** Global work wasted ,**HCM:** hypertrophic cardiomyopathy ,**HF:** heart failure ,**HK:** Hong Kong ,**HR:** Heart rate ,**HRs:** hazard ratio, **HRV:** Heart Rate Variability , **hsTnT:** High-Sensitivity Troponin T,**HTN:** Hypertension ,**ICD:** Implantable cardioverter defibrillator ,**ICU:** Intensive care unit ,**IHD:** Ischemic heart disease ,**IST:** Inappropriate sinus tachycardia ,**LA:** Pulmonary artery pressure ,**LC:** Long covid ,**LF:** Low frequency **,LGE:** Late gadolinium enhancement ,**LHR:** Left heart ratio ,**LV GLS:** Left ventricular Global longitudinal strain ,**LVEDV:** Left Ventricular End-Diastolic Volume, **LVEF:** Left ventricular ejection fraction ,**LVH:** Left ventricular hypertrophy **,LVMI:** Left ventricular mass index ,**MACE:** Major adverse cardiovascular events ,**MACE:** Major adverse cardiovascular events , ,**MBF:** Myocardial blood flow ,**MFR:** Myocardial flow reserve ,**MI:** Myocardial infarction, **MRI:** Magnetic resonance imaging ,**NR:** Not reported, **NSVT:** Non- sustained ventricular tachycardia , **NT-proBNP:** N-terminal pro-B-type Natriuretic Peptide, **NYHA:** New York Heart Association ,**PASC-CVS:** Post acute sequelae of covid – cardiovascular system ,**PCr/ATP:** Phosphocreatine / Adenosine Triphosphate ratio, **PCR:** Polymerase chain reaction, **PCS vs non-PCS:** Post-COVID Syndrome vs non-Post-COVID Syndrome, **PE:** pulmonary embolism ,**PET:** positron emission tomography ,**PH:** pulmonary hypertension ,**PHQ-9**: Patient Health Questionnaire-9 (depression),**proBNP:** Pro B type natriuretic peptide ,**PVC:** premature ventricular contraction, **QRS:** QRS complex (ventricular depolarisation on ECG),**RAT:** rapid antigen test ,**RMSSD:** Heart Rate Variability metrics: Root Mean Square of Successive Differences, **RV GLS:** right ventricular global longitudinal strain ,**RVEF:** right ventricular ejection fraction, **RVLS:** right Ventricular Longitudinal Strain, **SDNN:** Heart Rate Variability metrics: Standard Deviation of NN intervals; ,**SGRQ:** St George’s Respiratory Questionnaire (quality of life in respiratory disease), **SOB:** shortness of breath , **sPAP:** Systolic Pulmonary Artery Pressure, **SPECT/CT:** SPECT combined with CT,**SPECT:** Single Photon Emission Computed Tomography, **SSS:** Summed Stress Score (nuclear cardiology),**STE:** Speckle tracking echocardiography , **T1:** Relaxation time – CMR tissue characterisation; reflects fibrosis infiltration ,**T2:** Relaxation time- CMR tissue characterisation; reflects edema or inflammation ,**TAPSE:** Tricuspid Annular Plane Systolic Excursion, **TTE:** Transthoracic Echocardiography ,**UKB:** UK Biobank ,**VO2:** volume of oxygen
