# Supplementary material for: Long-term cardiovascular impact of COVID-19 among hospitalised and non-hospitalised populations: a narrative synthesis review
Source: Front Cardiovasc Med. 2026 May 7;13:1741293. doi: 10.3389/fcvm.2026.1741293 (PMC13190603; doi:10.3389/fcvm.2026.1741293)
Supplement: Supplementary file 2 [file Datasheet1.zip › Supplementary Table 2c.docx]

**Supplementary Table 2c: Characteristics of included studies (Outcome Characteristics)**

| **Reference** | **Primary outcome of Interest** | **Secondary outcome of Interest** | **Results** |
| --- | --- | --- | --- |
| 22 | Feasibility of virtual in-home monitoring; incidence/frequency of arrhythmia | % QRS complexes with supra/ventricular ectopic beats | 7/16 detected by AI (S-patch EX) to have cardiac arrhythmias but not clinically symptomatic. Patients with arrhythmias had a higher occurrence of SVT |
| 23 | Incidence of new/exacerbated cardiac diagnoses post-COVID; 1 death among referrals | Association of symptoms with COVID strain | 23% heart-related causes; 14% due to COVID (11% new, 4% worsening) among referrals |
| 77 | LV measurements, EF, EDV, GLS, LA volume, PA pressure; dyspnea common | Nil reported | Few echo abnormalities after mild COVID; low LV GLS and abnormal RV GLS common; severe abnormalities <1%; more frequent in men |
| 78 | HRV changes in females >3 months post-infection | Autonomic function survey; QoL score | Lower HRV indices and higher COMPASS-31 scores in symptomatic groups; QoL worse (p<0.05) |
| 24 | Occurrence of new COVID-related CV diagnoses and MACE over 1 year | MACE (ACS, stroke, CV death); cardiac dysfunction detected by imaging | LA dilation more common in new CV dx (p=0.017); abnormal PFT associated with new CV diagnosis (p=0.035); NYHA class linked to symptom non-improvement (p=0.008) |
| 25 | Incidence and HR of CVD and death acute & post-acute | Nil reported | Acute phase HR for CVD 4.3-5.0; all-cause mortality HR 67.5-81.1 vs controls. Post-acute HR for CVD 1.3-1.4; mortality HR 4.5-5.0 |
| 26 | HRV, HR, BP, exercise tolerance | Nil reported | Non-COVID group covered greater 6MWT distance |
| 27 | RV free wall longitudinal strain, LV GLS, arrhythmias | Comprehensive echo parameters; fatigue via Chalder scale | COVID worse RVLS (mean diff 1.5 pp; 95%CI -2.6 to -0.5; P=0.005), lower TAPSE and CI; diastolic dysfunction OR 2.4 (P=0.001); arrhythmias in 27% (PVC 18%, NSVT 5%) |
| 28 | Myocardial edema and LGE by CMR | None reported | 58% abnormal CMR; edema 54%, LGE 31%; native T1/T2/ECV elevated (p=0.002, <0.001, 0.002) |
| 29 | CMR-confirmed non-ischemic cardiac injury (LGE/myocarditis) | Extensive CMR metrics (LVEF, LVEDV, T1/T2/ECV, RVEF) and CT pneumonia extent | 52.9% had non-ischemic injury; LGE in 52.1%, myocarditis in 8.3%; hospitalized had lower RVEF (p=0.001) |
| 30 | Incidence of cardiac symptoms 1 year post-infection | VO2 max, 6MWT (exercise capacity) | No impairment in exercise capacity; normal LVEF and GLS vs controls; no significant cardiac dysfunction detected |
| 31 | Cardiac problems ~4 months post-recovery; sex differences | 10-year ASCVD risk; QoL symptoms | Echo abnormalities 16.7% men vs 9.7% women (p=0.1); benign arrhythmias ~45% both sexes; preexisting ASCVD higher in men (21.8% vs 6.1%, p<0.001); SCORE2 risk higher in older men (p<0.001) |
| 32 | Prevalence of cardiopulmonary long-term symptoms after severe COVID-19 | Predictors of Long-COVID; pulmonary function | ICU admission (p=0.039), longer hospital stay (p=0.001), higher NT-proBNP (OR 1.5; p=0.026) predicted Long-COVID; reduced GLS 11%; exertional dyspnea associated with impaired pulmonary/cardiac function (p-values 0.026-0.04) |
| 33 | Physical & psychological symptoms; fatigue; cardiac function at 6 months | QoL questionnaires (PHQ-9, GAD-7, SGRQ, EQ-5D-5L) | Fatigue in 28%; GLS median 15%; 68% had GLS >’16% (impaired); no significant difference in GLS between fatigued and non-fatigued (p=0.34) |
| 34 | Cardiovascular sequelae and mental health; QoL | Echo measures and correlations with pulmonary injury and mental health | Reduced LVEF (<50%) in ~9%; diastolic dysfunction ~28%; elevated sPAP ~28%; reduced FAC ~34%; symptom burden correlated with worse QoL and mental health |
| 35 | CMR prevalence of CV pathology; Long COVID vs non-Long COVID | CV PASC characterization; cardiac function vs controls | Majority moderate symptoms; RVEF reduced in cases vs controls (51.0% vs 53.2%, p=0.012); myocarditis 8%, Takotsubo 2% |
| 36 | Myocardial energetics (PCr/ATP), structure, perfusion, fibrosis | QoL and activities of daily living impact | No impairment in PCr/ATP or LV/RV function vs controls |
| 79 | Diagnosis of cardiovascular disorders (myocardial injury, PE) in CV long COVID | Duration of long COVID, palpitations, prognosis | 27% had CV disorder; myocardial injury 15%, PE 8%; severe condition and in-hospital cardiac events higher in CV disorder group; severe condition OR 5.79 (p=0.017), in-hospital events OR 8.08 (p=0.021) |
| 37 | Cardiomyopathy, arrhythmias, IHD, PH, HF, ECG abnormalities post-COVID | Nil reported | Descriptives show worsening BP, RR, increase in arrhythmias, LVH, decline in LV/RV function and rising PASP over time |
| 69 | Symptom duration and incidence vs cardiac function | Cardiovascular dysfunction and imaging abnormalities | Descriptive only: long-term CV symptom patients had higher BP, HR, weight vs pre-infection |
| 38 | Imaging finding’s cardiac function; myocarditis diagnosis | Patients meeting myocarditis criteria | 2% definitive myocarditis; 14% abnormal CMR without full criteria; most biomarkers (hsTnT, NT-proBNP) normal |
| 39 | Myocardial dysfunction (reduced GLS and LVEF) post-COVID vs controls | Time since infection, hospitalization, GLS as variables | Post-COVID had lower LVEF and GLS vs controls; 18.4% had abnormal GLS; GLS independent predictor of myocardial injury |
| 40 | Cardiac changes post-COVID vs post-vaccination; myocarditis/pericarditis/ischemia | Myocardial changes post-virus and post-vaccine | Higher prevalence of cardiac findings in post-vaccination group vs post-COVID despite smaller sample; differences in myocarditis presentation |
| 80 | Presence of CMD | ECG, exercise tolerance, coronary function | PET showed decreased MFR 1.34 confirming CMD |
| 81 | First occurrence of CV events (dysrhythmia, IHD, thrombotic, stroke, HF, myocarditis) by care setting | Analyses per CVE subgroup and hospitalization setting | Large cohort (n=1,357,518). ICU aHR 1.80 (95%CI 1.71-1.89); non-ICU hosp aHR 1.28 (1.24-1.33) vs outpatients; hospitalization increased subsequent CVE risk (aHRs up to 3.47) |
| 41 | Association between infective endocarditis and COVID-19 | Echo results | Pathological abnormalities identified (descriptive) |
| 42 | CV abnormalities by TTE at 4-8 weeks post-COVID; HF symptoms and recovery | Factors affecting evolution of pathological findings; stratified groups | Impaired LVF in 35, increased sPAP in 51, DD in 66, pericardial effusion in 23; TTE alterations largely improved over follow-up; correlations LV-GLS with lung injury and CRP (p<0.001); 26% had CV complications |
| 43 | Persistence of RV and LV myocardial function/hemodynamics using routine and STE | Groups stratified by clinical acuity; symptoms and QoL correlations | Decreased LV longitudinal strain vs controls; RV function improved over time; persistent LV dysfunction in 25%; abnormal RV strain linked to dyspnea |
| 82 | Incidence of CV symptoms and disease (hypertension) | Factors affecting severity of CV symptoms; risk factors identified | Compared to non-CV symptom patients, ORs: SOB 3.52, dizziness 2.04, GI symptoms 2.03; Women OR 2.08; young people OR 2.56; prehypertension OR 1.91; HTN OR 2.29; prior CV disease OR 1.86; diabetes OR 2.14 |
| 44 | Variation in HR and HRV measures post-COVID | Nil reported | SDNN and RMSSD higher in post-COVID (p<0.05); SDNN>60ms OR 2.4 (95%CI 1.2-12.8); RMSSD>40ms OR 2.5 (95%CI 1.4-9.2) after adjustment |
| 45 | Prevalence of cardiovascular dysautonomia (OH/POTS) in post-COVID | AI model to identify HRV measure for short-term ECG | 15.21% CV dysautonomia; RMSSD lower in post-COVID (13.9±11.8 vs 19.9±19.5 ms; p=0.01). HRV decreased with increasing COVID severity (p<0.0001). Common symptoms: dyspnea 17.4%, palpitations 16.3%, dizziness 14.1%, fatigue 11.9% |
| 46 | Prevalence of any new cardiac damage (NCA) post-COVID; symptomatic vs non-symptomatic | CVRF and comorbidities prevalence | 77.5% continued symptoms at median 96 days; NCA in 27.5% (pericardial effusion 11.95%); NCA patients older, more smokers, more CAD, stroke/TIA; higher low LVEF in hospitalized |
| 74 | Myocardial function via 2D-STE (GLS, myocardial work) in symptomatic recovered patients | Association between illness severity and myocardial function; CPX testing | VO2peak median 53% predicted; GWW higher and GWE lower in critical subgroup (p=0.01, p=0.03); illness severity independent predictor of GWW and GWE |
| 20 | Baseline and follow-up cardiac function in symptomatic v asymptomatic post-COVID | Symptoms persistence and imaging markers | Baseline median 109 days: 73% had cardiac symptoms; at ~329 days 53% persistent symptoms; symptomatic had higher HR and contrast uptake; diffuse myocardial edema more in those persistently symptomatic; female gender and baseline diffuse involvement predicted persistent symptoms |
| 47 | Cardiac sequelae and ECG/Echo changes at follow-up | Structural changes vs guidelines | Retrospective: arrhythmia 10%; prospective: repolarization abnormalities 24%, conduction 8%, arrhythmias 6% at follow-up |
| 83 | Incidence of cerebrovascular, arrhythmia, inflammatory/ischemic heart disease, thromboembolic events, MACE, death | Mortality rate higher in COVID group vs control | HRs: stroke 1.62, AF 2.41, myocarditis 4.41, IHD 2.81, HF 2.30, PE 2.65; MACE HR 1.87 |
| 70 | MACE composite and components as primary endpoint | Statin use, mortality, readmission, NYHA class | Prior CVD predicted MACE (HR 2.95, p=0.005); statin use associated with reduced MACE (HR 0.83, p=0.044), reduced stroke and revascularization |
| 84 | CMR LGE prevalence; LV function and strain parameters | Reproducibility and timing (median 48 days post-diagnosis) | LGE in 28%; normal LV systolic function overall; strain parameters lower in COVID survivors vs controls |
| 19 | Organ dysfunction/inflammation vs controls (multi-organ assessment) | Signs/symptoms per organ | No difference in LV EF/EDV or myocarditis overall; hospitalized had increased LV EDV (p=0.04); myocarditis incidence higher in severe vs moderate (p=0.027) |
| 88 | MRI tissue characterization in suspected myocarditis/cardiomyopathy referrals | T1/T2/LGE/strain differences | Suspected myocarditis patients had increased T1/T2/LGE and decreased strain vs non-myocarditis; LVEF and RVEF lower in COVID sufferers |
| 48 | Prevalence and characteristics of de novo cardiovascular findings (DNCFs) | Clinical variables associated with DNCFs from medical records | 18% of hospitalized had intra-COVID CV complications; DNCFs in 25.2% (17% rhythm disorders, 8% ventricular dysfunction); myocarditis in 6, CAD in 5, valvular disease in 2 |
| 71 | Long-term risk of multi-organ complications and mortality in older adults (cardiac focus) | Various outcomes (stroke, HF, MI, arrhythmia) | Elevated hazard ratios (HRs) were observed in older adults for major CVD in the UK Biobank (UKB: HRs 1.4) and Hong Kong cohort (HK: HRs 1.2). For MI, HRs were 1.8 (UKB) and 1.2 (HK); for HF, 1.6 (UKB) and 1.8 (HK); and for CHD, 1.5 (UKB) and 1.2 (HK) |
| 49 | RR interval variability (HRV) in long COVID | Nil reported | Reduced global HRV, increased sympathetic markers (LF, LF/HF), decreased parasympathetic (RMSSD, HF); reduced sympathovagal balance vs controls |
| 50 | Myocardial injury and subclinical dysfunction via CMR (T1/T2/LGE) | Comparison by illness severity | In 30 patients, 53.3% abnormal CMR; edema 40%, LGE 33.3%; severe cases had higher LGE and edema; T1 and T2 significantly higher vs HCs (p<0.0001 and p=0.04) |
| 85 | Cardiac function and measurements pre- vs post-COVID (LA/LV volumes, EF, strain) | Symptoms pre/post COVID | Paired analyses: reduced LVEF (p=0.049) and TAPSE (p=0.046) post-COVID but overall no clinically significant changes vs baseline; subgroup analyses by symptom status non-significant |
| 51 | Cardiac remodeling/ dysfunction after hospitalization for COVID (HTN vs non-HTN) | Ventricular measurements and functions | Hospitalized pts had LV concentric remodeling, grade I DD, mild longitudinal systolic function decrease vs controls |
| 52 | Global longitudinal strain (GLS) and myocardial work indices post-COVID | Global Work Index, GWW, GWE etc. | ~48.3% of post-COVID syndrome patients had subtle systolic dysfunction (GLS < -20%) |
| 53 | Prevalence of myocardial ischemia in long COVID (SPECT MPI) | Exercise tolerance, coronary circulation | Long COVID had higher abnormal SSS (p<0.05); CRP, SPECT LHR, and long COVID were independent predictors of ischemia; long COVID strongest predictor (p<0.001) |
| 54 | ECG/Echo/MRI correlates of myocardial dysfunction in non-hospitalized patients | Nil reported | QRS fragmentation and arrhythmias may indicate myocardial damage; descriptive suggestion for long-term monitoring |
| 55 | Cardiac function and structural measurements post-COVID (first 3 months) | Symptoms persistence | >70% had ventricular dysfunction in first 3 months; 40% persistent symptoms; decreased TAPSE (p<0.001), increased LV and RV GLS (p<0.001) in moderate-severe episodes |
| 56 | Incidence of IST, HR and NN intervals | Impaired QoL | IST pts had more environmental allergy (25% vs 0%, p=0.01); palpitations 90% vs 5% PCS+IST vs recovered (p<0.001); 6MWT median 392±83 m (~60% predicted) |
| 89 | Echo results in vaccinated vs unvaccinated; relationship with long-COVID | Comorbidities; vaccination and persistent symptoms | Vaccination associated with lower cardiac injury (1.35% vs 4.11%; adj OR 0.33; p=0.01) and fewer persistent symptoms (10.7% vs 18.3%; adj OR 0.52; p<0.001) |
| 90 | Risks and 1-year burdens of incident CVD after COVID | Incident CVD vs contemporary and historical controls; sensitivity analyses | Dysrhythmia composite HR 1.69 (1.64-1.75); IHD composite HR 1.66 (1.52-1.80); other CV disorders HR 1.72 (1.65-1.79); MACE HR 1.55 (1.50-1.60); burdens per 1000 reported |
| 57 | Coronary artery microvascular resistance / flow (AMR) in COVID pts | Assoc with demographic/clinical factors | Mean AMR higher in COVID (295 vs 266; p=0.002); COVID associated with increased AMR (OR 3.32; 95%CI 1.50-7.60; p=0.004) |
| 86 | ECG changes and septal flash (echo) clustering | Nil reported | Clusters: LBBB, septal flash, presystolic wave, POTS observed (descriptive) |
| 58 | Myocardial blood flow in PASC-CVS patients | Pathology markers (cholesterol, CRP) differences vs non-COVID | Resting MBF higher in PASC-CVS (1.29±0.27 vs 1.08±0.20 ml/g/min; p≤0.024); MFR lower (1.97±0.54 vs 2.27±0.43; p≤0.031); longitudinal MBF gradient present (p<0.0001) |
| 59 | In-hospital clinical characteristics & predictors of PCS | HR, exercise tolerance, echo results (PCS vs non-PCS) | MRC dyspnea higher in PCS (pre-discharge p=0.005; visit2 p=0.002); diastolic LV changes at visit2 e' (p=0.025), E/e' (p=0.020) |
| 60 | Evidence for myocardial injury by CMR (LGE) | Cardiac function and blood markers | LGE in 30% of patients; LGE-positive had decreased LV GCS, RV GCS, RV GLS vs LGE-negative (p<0.05) |
| 61 | Incidence of CV event within 30 days after hospitalization | Mortality and factors associated with CV events | 62 patients (15.7%) had CV events on follow-up; 30-day mortality 20.3%; independent predictors: HTN, intubation, age>75 |
| 62 | Correlation of CV risk (CVR) with symptoms, respiratory changes, hospitalization | CVR and acute disease severity | High/very-high CVR associated with increased hospitalization, ICU admission, development of post-COVID, and larger post-COVID disease burden (p<0.0001) |
| 75 | 2D-STE detection of subclinical myocardial dysfunction; LV-GLS as predictor of MACE | Long-COVID prevalence and prognosis | Subclinical dysfunction in 27% (normal LVEF) and 34% overall; LV-GLS impairment associated with long-term MACE (AUC 0.73); LV-GLS & NYHA>1 independent predictors; long-COVID (44%) not linked to worse prognosis |
| 63 | Echo findings: LVEF, LV GLS, RV function across waves | Wave comparison (wave1 v wave2) | Reduced LVEF in 27%, reduced LV GLS in 30%; RV dysfunction (TAPSE 14%, RV FAC 18%, RV strain 43%); no significant difference wave1 v wave2 for RV dysfunction (p=0.63); CRP and ICU admission higher in wave1 |
| 64 | Risk of MACE and AMI post-SARS-CoV-2 vs unexposed (by vaccination status & severity) | Incident MACE by hospital status and vaccination | aHR overall 1.34 (1.22-1.46); hospitalized aHR 3.81; ICU aHR 6.25; attributable fraction 7.04% for CV events |
| 65 | Sex differences in ECG/echo/biochemistry post-COVID | Nil reported | Males more ECG abnormalities (27.3% vs 19.2%, p=0.004); females higher arrhythmia and HR (p<0.001 & p=0.018); sex differences in lipid profile and BP measures (p<0.001) |
| 76 | Subclinical myocardial injury during hospitalization and follow-up recovery | Troponin, NT-proBNP changes | 46.4% had subclinical injury during hospitalization; 46.2% persisted LV dysfunction at follow-up; TAPSE and RVLS improved significantly (p<0.001); GLS did not improve (p=0.6); lower GLS, TAPSE, RVLS vs controls (p<0.01) |
| 66 | Coronary sinus flow and myocardial perfusion reserve vs controls | Stress response comparisons (HCM, controls) | COVID group had greater resting CSF and blunted CSF increase under stress (43% vs controls 279%); lower myocardial perfusion reserve and RV EF lower in COVID |
| 67 | Subacute/chronic myocarditis post-COVID | Response to corticosteroids; viral persistence | Clinical improvement with corticosteroids common; SARS-CoV-2 RNA persisted up to 18 months in myocardium (case series) |
| 87 | Echo differences: long COVID vs control (LA diam, LVMI, diastolic markers) | NR | Long COVID pts had higher age, metabolic age, LA diameter, LVMI, A velocity, E/E' and lower E/A vs controls (p<0.05) |
| 72 | Rates of death, readmission, multiorgan dysfunction post-discharge; age-stratified risks | NR | Rate ratio of MACE greater for people aged >70 vs matched controls; survival analyses stratified by hospitalization and time period |
| 73 | All-cause mortality in the year following COVID testing; time-stratified risk analysis | NR | Time‑stratified analysis showed an increased risk of all‑cause mortality within the first 30–60 days after COVID‑19 infection, particularly among hospitalised individuals with high ASCVD risk. Mortality risk returned to baseline thereafter, with no excess risk at 1‑year follow‑up |
| 68 | LV GLS and exercise capacity changes over time in athletes/recovered pts | CPET max power; longitudinal changes t0->t1 | LV GLS improved (p<0.001); max CPET power higher at t1 (p=0.009); mixed effects models controlled for confounders |

**6MWT**: six minute walk test ,**ABPM**: ambulatory blood pressure monitoring ,**ACS:** acute coronary syndrome ,**aHR:** adjusted hazard ratio**, AMR:** absolute microvascular resistance ,**ASCVD risk**: Atherosclerotic Cardiovascular Disease Risk, **BP**: Blood pressure, **CHD:** Coronary heart disease ,**CMD:** Coronary microvascular dysfunction , **CMR:** Cardiac Magnetic Resonance, **CMR:** Cardiac magnetic resonance ,**COMPASS-31:** Composite Autonomic Symptom Score (31-item questionnaire),**COVID-19**: Coronavirus disease 2019,**CPET:** Cardiopulmonary exercise test ,**CRP:** C-reactive protein ,**CV:** Cardiovascular ,**CVD**: Cardiovascular disease,**CVE:** Cardiovascular event ,**ECG:** Electrocardiogram ,**ECV:** Extracellular volume ,**EDV**: End-diastolic volume ,**EF:** ejection fraction ,**EQ-5D-5L**: EuroQol 5-Dimensions 5-Level (health-related quality of life),**GAD-7**: Generalized Anxiety Disorder-7 (anxiety),**GLS:** Global longitudinal strain ,**GW:** Global work ,**GWE:** Global work efficiency ,**GWW:** Global work wasted ,**HCM:** hypertrophic cardiomyopathy ,**HF:** heart failure ,**HK:** Hong Kong ,**HR:** Heart rate ,**HRs:** hazard ratio, **HRV:** Heart Rate Variability , **hsTnT:** High-Sensitivity Troponin T,**HTN:** Hypertension ,**ICD:** Implantable cardioverter defibrillator ,**ICU:** Intensive care unit ,**IHD:** Ischemic heart disease ,**IST:** Inappropriate sinus tachycardia ,**LA:** Pulmonary artery pressure ,**LC:** Long covid ,**LF:** Low frequency **,LGE:** Late gadolinium enhancement ,**LHR:** Left heart ratio ,**LV GLS:** Left ventricular Global longitudinal strain ,**LVEDV:** Left Ventricular End-Diastolic Volume, **LVEF:** Left ventricular ejection fraction ,**LVH:** Left ventricular hypertrophy **,LVMI:** Left ventricular mass index ,**MACE:** Major adverse cardiovascular events ,**MACE:** Major adverse cardiovascular events , ,**MBF:** Myocardial blood flow ,**MFR:** Myocardial flow reserve ,**MI:** Myocardial infarction, **MRI:** Magnetic resonance imaging ,**NR:** Not reported, **NSVT:** Non- sustained ventricular tachycardia , **NT-proBNP:** N-terminal pro-B-type Natriuretic Peptide, **NYHA:** New York Heart Association ,**PASC-CVS:** Post acute sequelae of covid – cardiovascular system ,**PCr/ATP:** Phosphocreatine / Adenosine Triphosphate ratio, **PCR:** Polymerase chain reaction, **PCS vs non-PCS:** Post-COVID Syndrome vs non-Post-COVID Syndrome, **PE:** pulmonary embolism ,**PET:** positron emission tomography ,**PH:** pulmonary hypertension ,**PHQ-9**: Patient Health Questionnaire-9 (depression),**proBNP:** Pro B type natriuretic peptide ,**PVC:** premature ventricular contraction, **QRS:** QRS complex (ventricular depolarisation on ECG),**RAT:** rapid antigen test ,**RMSSD:** Heart Rate Variability metrics: Root Mean Square of Successive Differences, **RV GLS:** right ventricular global longitudinal strain ,**RVEF:** right ventricular ejection fraction, **RVLS:** right Ventricular Longitudinal Strain, **SDNN:** Heart Rate Variability metrics: Standard Deviation of NN intervals; ,**SGRQ:** St George’s Respiratory Questionnaire (quality of life in respiratory disease), **SOB:** shortness of breath , **sPAP:** Systolic Pulmonary Artery Pressure, **SPECT/CT:** SPECT combined with CT,**SPECT:** Single Photon Emission Computed Tomography, **SSS:** Summed Stress Score (nuclear cardiology),**STE:** Speckle tracking echocardiography , **T1:** Relaxation time – CMR tissue characterisation; reflects fibrosis infiltration ,**T2:** Relaxation time- CMR tissue characterisation; reflects edema or inflammation ,**TAPSE:** Tricuspid Annular Plane Systolic Excursion, **TTE:** Transthoracic Echocardiography ,**UKB:** UK Biobank ,**VO2:** volume of oxygen
